# Greenland ice mass loss during the Younger Dryas driven by Atlantic Meridional Overturning Circulation feedbacks

**DOI:** 10.1038/s41598-018-29226-8

**Published:** 2018-08-09

**Authors:** Eleanor Rainsley, Laurie Menviel, Christopher J. Fogwill, Chris S. M. Turney, Anna L. C. Hughes, Dylan H. Rood

**Affiliations:** 1 School of Geography, Geology and the Environment, University of Keele, Staffordshire, UK; 2 Climate Change Research Centre and PANGEA Research Centre, School of Biological, Earth and Environmental Sciences, University of New South Wales, Sydney, NSW 2052 Australia; 3 Department of Earth Science, University of Bergen and Bjerknes Centre for Climate Research, Bergen, 5007 Norway; 4 Department of Earth Science and Engineering, Imperial College London, South Kensington Campus, London, SW7 2AZ UK; 5 Scottish Universities Environmental Research Centre, East Kilbride, G75 0QF UK

## Abstract

Understanding feedbacks between the Greenland Ice Sheet (GrIS) and the Atlantic Meridional Overturning Circulation (AMOC) is crucial for reducing uncertainties over future sea level and ocean circulation change. Reconstructing past GrIS dynamics can extend the observational record and elucidate mechanisms that operate on multi-decadal timescales. We report a highly-constrained last glacial vertical profile of cosmogenic isotope exposure ages from Sermilik Fjord, a marine-terminating ice stream in the southeast sector of the GrIS. Our reconstruction reveals substantial ice-mass loss throughout the Younger Dryas (12.9-11.7 ka), a period of marked atmospheric and sea-surface cooling. Earth-system modelling reveals that southern GrIS marginal melt was likely driven by strengthening of the Irminger Current at depth due to a weakening of the AMOC during the Younger Dryas. This change in North Atlantic circulation appears to have drawn warm subsurface waters to southeast Greenland despite markedly cooler sea surface temperatures, enhancing thermal erosion at the grounding lines of palaeo ice-streams, supporting interpretation of regional marine-sediment cores. Given current rates of GrIS meltwater input into the North Atlantic and the vulnerability of major ice streams to water temperature changes at the grounding line, this mechanism has important implications for future AMOC changes and northern hemisphere heat transport.

## Introduction

In today’s warming climate, the persistent North Atlantic cooling anomaly off the southeast coast of Greenland is thought to be caused by the accelerating input of meltwater from the Greenland Ice Sheet (GrIS) and considered an indicator of a weakening of the Atlantic Meridional Overturning Circulation (AMOC)^1,2^. Modelling studies suggest that increased freshwater input from Greenland has the potential to slow down AMOC in the future^3^, thus substantially weakening heat transport to the North Atlantic and potentially driving positive ice sheet-ocean feedbacks^4^.

Unfortunately, contemporary observations of the GrIS and North Atlantic are too short (<100 years) to inform on our understanding of long-term trajectories and feedbacks over multi-decadal to centennial timescales. Periods of large-scale rapid climate reorganisation in the recent geological past offer considerable potential for understanding ice-sheet-ocean dynamics, thereby extending the observational window. The Younger Dryas (YD, broadly coincident to Greenland Stadial 1, ~12.9-11.7 ka^5^) was a period of sustained Northern Hemisphere atmospheric cooling of up to 10** °**C ^6,7^ that is considered to have been initiated by freshwater input into the Arctic Ocean and/or North Atlantic from the expanded Laurentide Ice Sheet^8^. In contrast to widespread terrestrial glacier expansion in Europe during the YD^9^, ice streams and local glaciers in southern Greenland may have reacted in antiphase to atmospheric temperature changes^9^; local terrestrial glaciers near Scorsby Sund, east Greenland, show evidence of Younger Dryas standstill^10^, whilst reconstructions of the Jakobshavn Isbrae and Kangerlussuaq ice streams (west and east Greenland respectively) suggest possible retreat^11–14^. However, this intriguing potential non-linear response is poorly constrained by current field data, and is not presently captured in ice sheet models, the latter perhaps owing to an underrepresentation of ocean processes^15^. These discrepancies are, however, consistent with suggestions of divergent ocean circulation behaviour across the North Atlantic, with steady or even decreasing marine ^14^C reservoir ages southeast of Greenland^16,17^ at a time of substantial increase across the wider North Atlantic^18^. Intriguingly, marine sediment cores from the southeast Greenland continental shelf appear to record sustained freshwater spikes from 12.8-12.0 ka that have been interpreted as a consequence of significant meltwater input into the North Atlantic possibly sourced from the GrIS^17,19^, but due to its fragmentary nature, terrestrial evidence from ice-free areas of the GrIs is at present inconclusive and contradictory. Thus, the YD may provide an extreme scenario that could allow atmospheric temperature effects to be disentangled from ice sheet-ocean interactions, and given current uncertainties, developing new and detailed terrestrial records of deglaciation is crucial.

**Figure 1:**
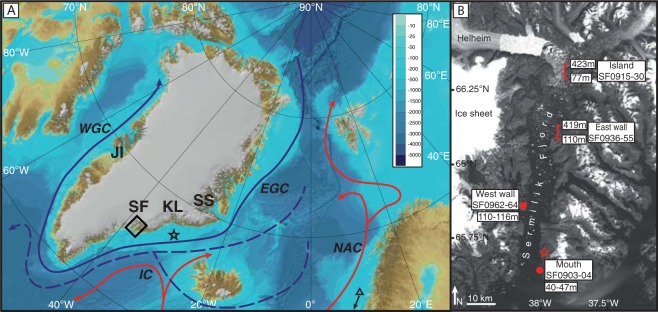
Location map. (**A**) Northeast Atlantic region with ocean currents in solid lines (IC: Irminger Current; NAC: Norwegian Atlantic Current; EGC: East Greenland Current; WGC: West Greenland Current) and return flow of NADW in dashed lines. Red and blue lines indicate warm and cold currents respectively. JI: Jakobshavn Isbrae; KL: Kangerlussuaq; SS: Scoresby Sund; SF: Sermilik Fjord. Box shows location of (**B**). Black star shows location of ocean cores referenced in text^16,17^. Triangle shows location (just south of this image) of ocean cores from. Bathymetry of ocean shown in inset scale in m b.s.l. Base map and bathymetry from https://www.ngdc.noaa.gov/mgg/bathymetry/arctic/ (Version 3.0 ref.^67^. (**B**) Locations and altitudes of *in situ* cosmogenic exposure ages in Sermilik Fjord. Location and altitudinal range of the Island (samples SF0915-30) and East wall (samples SF0936-55) vertical transects shown by red brackets. Discrete sample locations (West wall and Mouth) and altitudes shown by red dots. Red star shows location of basal radiocarbon dates from lakes^26^. Base Landsat image downloaded from Earth Explorer *(https://earthexplorer.usgs.gov/ USGS/NASA Landsat Program). Figure generated in Adobe Illustrator 2015 1.0 release (19.1.0).

Reconstructing the deglacial thinning and retreat of the GrIS during the YD has been traditionally hampered by relatively large chronological uncertainties and limited vertical profile constraints. Here we report the first three-dimensional reconstruction of deglacial thinning and retreat of a major GrIS palaeo ice stream from Sermilik Fjord, providing new insights into the complex three-dimensional dynamics of the Sermilik outlet and the wider southern GrIS (Fig. 1). Today, this fjord routes half of the ice mass from five of the major ice sheet basins^20^, making it highly sensitive to dynamic changes both at the North Atlantic margin and across the wider ice sheet. By developing a vertical profile of cosmogenic isotope exposure ages using Bayesian modelling we identify sustained drawdown of the Sermilik ice stream prior to rapid Holocene retreat at ~10.8 ± 0.3 ka^21^, providing new insights into past ice-sheet ocean interactions and inform on non-linear feedbacks between atmospheric temperature change, GrIS melt and AMOC changes.

Over 80 km long and up to 12 km wide, Sermilik (65.98°N, 37.85°W) is the largest fjord in southeast Greenland, with the classic steep walls and geomorphology of a former ice sheet outlet. The lower part of the fjord trends roughly north-south, whilst the upper 30 km coincides with a narrowing and shallowing of the fjord (Fig. 1B). Bathymetric data shows maximum depths of ~900 m, decreasing to 300–600 m in the upper part and with bathymetric lows of <200 m^21^. The marine-terminating Helheim Glacier (which drains into Sermilik Fjord, Fig. 1B) has amongst the highest recorded rates of glacier-velocity acceleration in present-day Greenland, and is critical to regional mass balance both today and in the past^22^. Today this outlet glacier terminates at the head of Sermilik Fjord, but glaciological reconstructions demonstrate that at the Last Glacial Maximum (~20 ka) outlet glaciers of the GrIS terminated off-shore on the continental shelf in deep cross-shelf troughs at depths of ~600 m^23^.

The rapid retreat of the GrIS outlet in Sermilik during the early Holocene^21^ coincided with the abrupt Northern Hemisphere atmospheric warming following the YD^24^. However, in order to fully understand the dynamical response of the ice stream to environmental change, it is important to determine the timing and magnitude of thinning as well as the rate of terminus or lateral retreat. Here we present the results of new *in situ* cosmogenic isotope analysis of vertical transects which we combine with published lateral transects up Sermilik Fjord^21^ and examine possible drivers of the dynamic changes we discover using transient experiments performed with an Earth system model.

## Results

### Cosmogenic isotope analysis

Glacial erratics and bedrock were sampled along two individual vertical transects (one from an island in the north of the fjord, and another on the east wall), between ~40–430 m above present day sea level in Sermilik Fjord, as well as two additional discrete sites at lower elevations (Fig. 1). Samples were analysed for the *in situ* cosmogenic nuclide ^10^Be, and exposure ages calculated using the Northeast North American production rate (see Methods; Tables S1 and S2)^25^. The highest samples imply that the ice sheet surface was over 400 m a.s.l before 13 ka, inferring the presence of a fully-grounded ice stream at this time, given the depth of the fjord and the flotation point of ice.

**Figure 2:**
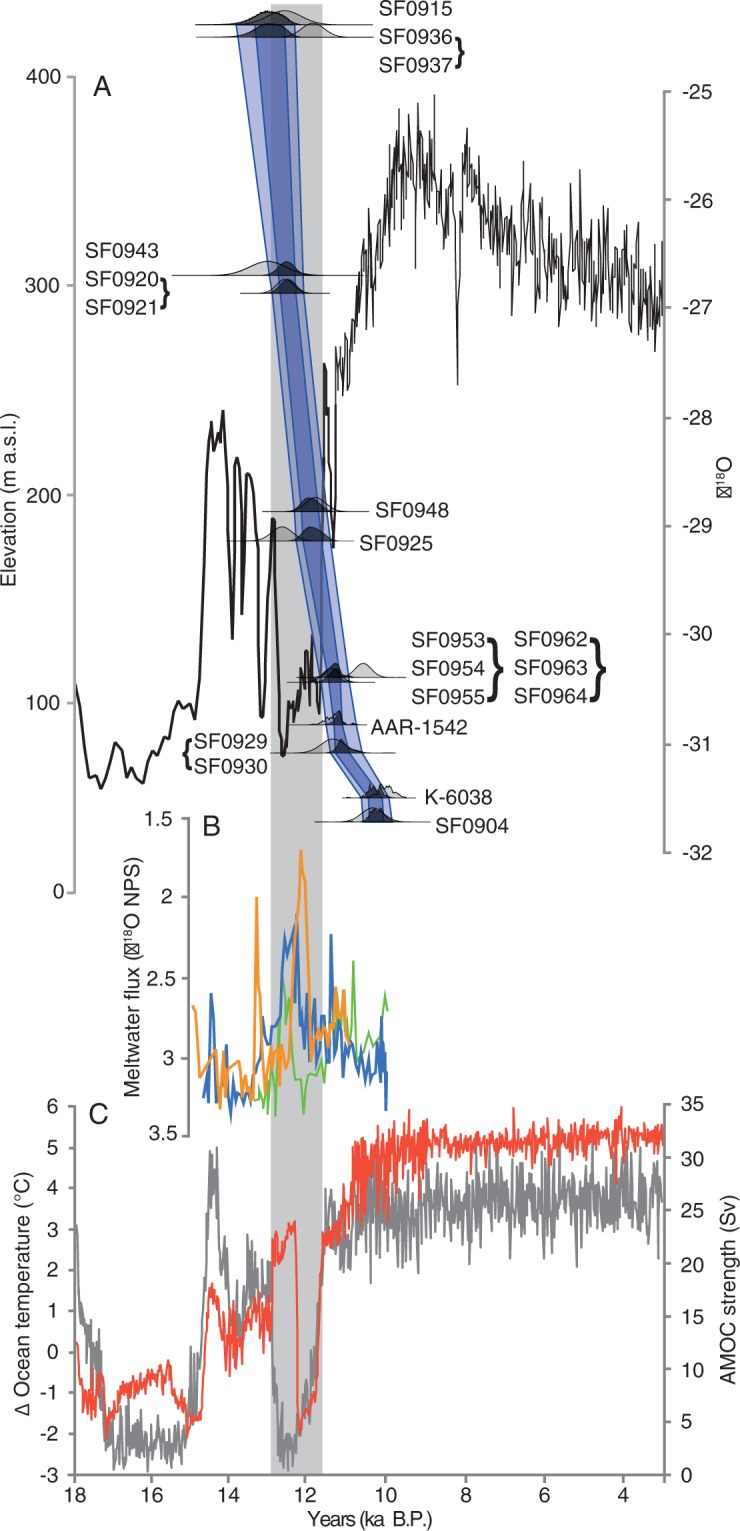
Sermilik Fjord thinning trajectory. (**A**) Age-modelled thinning trajectory of Sermilik Fjord from *in situ* cosmogenic exposure ages (SF numbers, brackets denote ^10^Be exposure ages combined into a single event by *C_Combine*) and two radiocarbon ages from basal lake sediments (AAR-1542 and K-0638)^26^. Dark blue envelope to 1 s.d., pale blue to 2.s.d. Light grey cumulative probability functions show raw ^10^Be exposure ages after outliers were omitted by *C_Combine* function (Table S2). Dark grey probability density functions show Bayesian modelled ages (Table S4). Plotted against δ^18^O curve from Renland Ice Core (black line)^24^. (**B**) Stable isotope data showing freshwater spikes from SE Greenland ocean cores JM96-1216/2-GC (green line), JM96-1215/2-GC (blue line) and JM96-1213/1-GC (orange)^17^. (**C**) Timeseries showing AMOC strength (Sv, grey line) and ocean temperature at 484- 694 m water depth off SE Greenland (62.5°N-67°N,40°W-36°W) (red line) as simulated in the LOVECLIM transient deglacial experiment^43^. YD (12.9-11.7 ka) marked with grey box. Constructed with OxCal 4.1 (ref.^27^) and Figure generated in Adobe Illustrator 2015 1.0 release (19.1.0).

To constrain the age-elevation history and produce a thinning model, we applied Bayesian modelling to the cosmogenic ages, in combination with two basal organic ^14^C ages from elevated lakes on the eastern side of Sermilik^26^, using OxCal 4.1^27^ (see Methods). This analysis reveals that ice stream drawdown was underway by 13.0 ± 0.4 ka (1 s.d.), and continued into the Holocene until 10.3 ± 0.2 ka (1 s.d.), prior to its retreat upstream, indicating the ice ungrounded and underwent surface lowering of ~376 m. This continued throughout the Younger Dryas, with ~280 m of the of the thinning occurring during this period (Fig. 2A). The timing of this significant mass loss agrees with the implied early YD retreat of Jakobshavn Isbrae^11,14,28^ as well as with the southeast Greenland freshwater spike at 12.8-12 ka^17^ (Fig. 2B), and suggests that late YD retreat of the Kangerlussuaq Fjord ice stream (east Greenland, previously known as Kangerdlugssuaq Fjord, and distinct from Kangerlussuaq Fjord in west Greenland)^12^ may have been preceded by a similar thinning. Crucially, the well-constrained trajectory we demonstrate here confirms previous suggestions of GrIs Younger Dryas ice retreat^9–11,14,28^. Our results appear to represent a coherent pattern of major ice stream downwasting and significant GrIS mass loss through a period of sustained atmospheric cooling in this important sector of the ice sheet (Fig. 2A).

### Exploring the drivers of Younger Dryas melt

Our observations raise the question: what could have driven substantial retreat of a major outlet glacier of the GrIS despite a marked drop in atmospheric temperature at the Younger Dryas? Rising sea levels can cause ice-stream instability and retreat; however, although global sea level was rising prior to and during the YD^29^, Greenland was undergoing substantial isostatic uplift following post-LGM ice-sheet retreat^30^, which may have proved an additional stabilising factor. Heightened seasonality (and therefore length of melt season) caused by the 11 ka peak in obliquity^31^ may also have contributed, but the YD expansion of a local terrestrial ice cap in the far north of Greenland^32^ implies that this effect was not strong enough to drive major ice stream retreat.

An alternative mechanism for negative mass balance could be reduction in precipitation in the accumulation zones of the GrIS as a result of sea ice expansion in the Northern Atlantic^33,34^. Reconstructions of Arctic sea ice at the time are limited and contradictory^35^, but modelling suggests that winter sea ice at 12 ka was expansive^36^ (Fig. S3). The GISP2 ice core shows a marked decrease in accumulation throughout the YD^37,38^, consistent with the idea of precipitation starvation (Fig. S4), but equally low levels of precipitation occurred during the LGM when the GrIS was expanding^39^. Furthermore, significant Arctic sea ice expansion during the Little Ice Age – including across the GrIS precipitation source of the Labrador Sea^40^ – apparently did nothing to inhibit glacial expansion^41^, which strongly suggests that sea-ice induced precipitation starvation alone could not have driven the YD mass loss.

**Figure 3:**
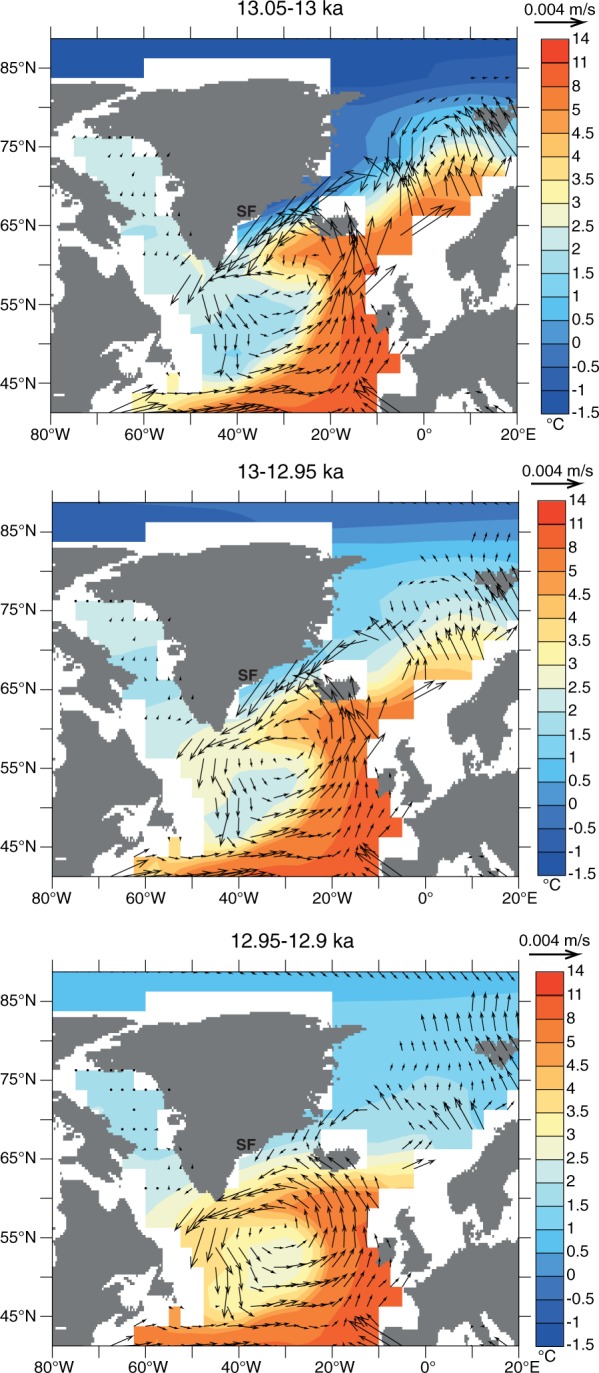
Modelled ocean dynamics. Simulated^43^ ocean temperature (shaded, °C) and currents (m/s) averaged over 484–693 m water depth immediately prior to the YD (top, 13.05-13 ka), at the onset of the event (middle, 13-12.95 ka), and during the early YD (bottom, 12.85-12.8 ka). (constructed using ferret http://ferret.pmel.noaa.gov/Ferret).

Although sea surface temperatures in southern Greenland were markedly depressed during the Younger Dryas^42^, the contrasting mass trends between the marine-terminating ice streams of the GrIS^11^ and terrestrial glaciers elsewhere in the Northern Hemisphere^9^ suggests oceanographic changes may have played a key role. To investigate potential feedback mechanisms between the GrIS and the North Atlantic oceanic response to a weakened AMOC, we analyse model outputs from a transient deglacial experiment performed with the Earth system model LOVECLIM^43^, where a weakening of the AMOC during the YD is simulated by a 0.25 Sv freshwater flux applied in the Arctic Ocean (175°W-95°W, 67°N-83°N) between 13 and 12.2 ka B.P (see Methods). Despite a marked simulated North Atlantic sea surface temperature decline and sea ice expansion (Fig. S3) during the early part of the YD, a sustained sub-surface ocean warming is simulated at mid-depth along the entire south-eastern Greenland margin (Figs 3 and S5). Before the YD event, North Atlantic Deep Water (NADW) formation at the confluence of the Greenland and Norwegian Seas (75–80°N) drives a subsurface poleward advection of relatively warm water to that region, as well as the southward advection of cold and dense NADW along the eastern margin of Greenland (Figs 1 and 3). Meltwater input into the Arctic at the beginning of the YD leads to the shutdown of NADW production at 12.8 ka (Fig. S3), which weakens the southward advection of cold subsurface waters through the Denmark overflow, and the advection of warm water from the Norwegian Atlantic Current (NAC) to the Norwegian Sea due to a reduced meridional density gradient in the North Atlantic and changes in the wind stress curl. This significantly reduces the strong east-west temperature gradient at depth, supporting regional palaeoceanographic reconstructions^17^ (Figs 2 and 3). This is a robust feature of North Atlantic Themohaline circulation in both earth system models of intermediate complexity and fully coupled atmosphere-ocean general circulation models^44^. Furthermore, within 100 years of the meltwater input, our results suggest the subsurface cyclonic circulation south of Iceland intensified, strengthening the Irminger Current and enhancing the subsurface advection of warm waters to the south of Greenland, as evidenced by marine micropalentological investigations from the Kangerlussuaq^17^ (Figs 1, 3 and S5). As a consequence, ocean temperatures below ~350 m increase markedly from 14 ka to 12.5 ka, with positive ocean temperature anomalies of ~3 °C and ~0.4 °C southeast (40–25°W, 63–70°N) and southwest (45–55°W, 60–68°N) of Greenland respectively, a depth comparable to those of cross-shelf troughs of the major ice streams (Figs 1, 3 and S5).

## Discussion

We propose that ice-mass loss from southern Greenland across the YD was driven by entrainment of warm sub-surface water in the cross-shelf troughs, leading to thermal erosion at the grounding line of southern GrIS marine-terminating outlets such as Sermilik and Kangerlussuaq^12^, consistent with the hypothesis that ocean circulation was more important than air temperature change in this region during the YD^15,17,19^. Increased stratification in the northeast Atlantic following the cessation of NADW formation increased the marine reservoir age in this region^18^, and the anomalously low marine reservoir age observed southeast of Greenland throughout the YD^16^ could be explained by such subsurface current reorganisations, supporting the interpretation of isotopic records from marine sediment cores from the North Atlantic off the SE GrIs^19^. Furthermore, micropalentological evidence suggests that recirculating intermediate depth North Atlantic water, advected via the Irminger Current, may have entered the Kangerlussuaq Trough during the YD chronozone, providing direct evidence for the subsurface current reorganisation we propose. Our model results suggest that the initial input of YD glacial meltwater into the North Atlantic from the Arctic and the Laurentide Ice Sheet triggered a positive ice sheet-ocean feedback, whereby a weakened AMOC – and the associated subsurface warming off the south Greenland shelf – helped sustain freshwater input into the North Atlantic, thus driving and potentially extending the Northern Hemisphere YD cooling. In our model experiments we do not add meltwater from the GrIs, however, as noted in other studies this may have enhanced this feedback mechanism along the eastern margin of the GrIs^17,19^.

Such a mechanism could have significant implications for future ice-sheet dynamics. A number of major outlets of the present-day southern GrIS have grounding lines at depths of 500–800 m b.s.l., including Helheim (which feeds into Sermilik Fjord), Kangerlussuaq (east Greenland) and Jakobshavn Isbrae^45^. The subsurface current reorganisation highlighted by our modelling study impacts depths of 150–900 m, which encompasses the grounding line depths near the margins of all these GrIS outlets^45^. If the contemporary observed AMOC weakening exacerbated by Greenland meltwater^1^ continues, the mechanism highlighted here may lead to warmer Atlantic Water being drawn towards southern Greenland in the future, significantly increasing subsurface ocean temperatures at the grounding lines of major outlets. This could drive enhanced thermal erosion of outlet glaciers, and further freshwater input into the North Atlantic over and above that caused by current atmospheric temperature changes. Given that these glaciers are already projected to contribute 19–30 mm to sea-level rise by 2200 (ref.^46^), it is vital that this feedback is included in ice-sheet modelling studies to constrain projections of future sea-level rise and northern hemisphere heat transport.

Our study reveals substantial Greenland ice stream collapse in the Younger Dryas during a period of considerable atmospheric and sea-surface cooling in the North Atlantic. Our finding supports the interpretation of marine sediment cores off the SE GrIS^17,19^, and suggest that ice sheet mass loss was driven by sub-surface warming driven by altered deep ocean convection in the Norwegian Sea and a subsequently weakened AMOC. This positive feedback mechanism could have significant implications for present-day GrIS/AMOC interactions^1^ and highlights complex and non-linear ice-sheet–atmosphere–ocean dynamics that must be incorporated into future projections.

## Methods

### Cosmogenic sampling strategy

Rock samples were collected for dating by *in situ* cosmogenic isotope analysis from ice-free areas along Sermilik Fjord, southeast Greenland during fieldwork in June/July 2009. Sample sites were chosen to span the greatest possible range within the time available for fieldwork, both geographically and attitudinally. Geological maps were consulted prior to sampling to ascertain ideal sampling areas for the quartz-rich rock types needed for ^10^Be measurement. Accessibility was also a key control when selecting sample areas; sites were accessed by boat and foot, so terrain and local conditions had to be considered. Where possible, samples were taken in bedrock-erratic pairs or triplets (i.e. bedrock-erratic or bedrock-erratic-erratic), to allow for any inconsistencies in glacial history to be detected. Samples were selected based on vegetation cover, potential for snow cover (samples in raised and exposed areas, less prone to snow accumulation were preferred) and their stability (e.g. erratic cobbles were sampled from areas outside of drift accumulation, and with weathering pedestals beneath them; erratics boulders were not sampled if they seemed precariously perched or liable to down-slope movement; bedrock was not taken from areas of higher weathering). Erratic samples were taken either as whole cobbles (up to ~20 cm) or as subsample of larger erratic boulders (up to 1 m), in which case the top surface was taken. Bedrock samples were taken from glacially streamlined surfaces. Samples were taken up to 12.5 cm thick, although thinner samples were preferred where possible. Samples from lower elevations were above the local marine limit (~40 m a.s.l. (ref.^47^)). Samples were taken along two vertical transects, from an island in the northern sector of the fjord (Island transect; samples SF0915-30) and from the east wall of the fjord (East wall transect; samples SF0936-55), as well as from two additional sites (West wall; SF0962-64 and Mouth; SF0903-04); see Fig. 1.

Samples were taken by hand using a four-pound lump hammer and chisel; all samples were labelled and photographed before being securely wrapped and relabelled, and detailed field notes on lithology, vegetation/snow cover and local geomorphology were made. Inclination measurements of the surrounding topography were measured to ascertain topographic shielding, using a handheld clinometer, every thirty degrees on the azimuth, with additional measurements taken if this missed significant features. Latitude, longitude and elevation readings were taken using a handheld GPS device (accurate to ±10 m in elevation).

### ^10^Be cosmogenic isotope extraction

Beryllium purification chemistry was undertaken at the University of Exeter Cosmogenic Isotope Laboratories. Chemistry followed protocols adapted from Ivy-Ochs^48^ and Ditchburn and Whitehead^49^, modified by C. Fogwill and T. Barrows/J. Stone, respectively. Samples were then ignited to Be oxide, mixed with Nb, loaded into copper cathodes, and ^10^Be/^9^Be measured by accelerator mass spectrometry (AMS) at Scottish Universities Environmental Research Centre (SUERC). AMS measurements were normalized to the NIST 27900 standard (NIST SRM4325 standard material with an assumed isotope ratio of 2.79 × 10^−11^). Procedural blank ratios were 5.16 × 10^−15^, 5.48 × 10^−15^, 3.65 × 10^−15^ and 1.83 × 10^−15^. Blank ratios were 0.5–10.7% of sample ratios (mean: 3.4%). The isotopic ratio of the procedural blanks for each batch of samples was subtracted from the sample ratio, with errors in sample and background propagated in quadrature. See Table S1 for sample details.

### Age calculation of cosmogenic exposures ages

Exposure ages were calculated using the CRONUS-Earth online age calculator^50^ (http://hess.ess.washington.edu), version 2.2, constants version 2.2.1, using the recently revised ^10^Be half-life (1.387 Ma refs^51,52^) and Be isotope ratio standardization of Nishiizumi *et al*.^53^. Age calculation assumed standard pressure, zero erosion and sample density of 2.62 g.cm^2^. Samples were scaled using the time-dependent Lal/Stone scaling scheme^54,55^, following similar studies and the recent analysis by CRONUS-Earth which shows the Lal scheme to provide a better fit to calibration date than the other, neutron-monitor based, schemes^56^; using other scaling schemes produces exposure ages which differ by 1–4% (~120–480 yrs). For our analysis, we use the production rate from the North American (NENA) calibration set (reference production rate at SLHL: 3.87 ± 0.19 atoms g^−1^ a^−1^ using the Lal/Stone time-dependent scaling scheme)^25^, in line with other studies in the area, but also because of the robustness of this calibration set for studies in Greenland^55,57^. NENA exposure ages for all samples are shown in Figure S1 and Table S2. Samples were also calculated using the global production rate – which is increasingly being shown as inaccurate in higher latitudes owing to its reliance on low-latitude, high-altitude calibration sites – which produced results systematically 11.37% older than those using the NENA production rate. Other regional production rates were also used: Northern Norway^58^ (mean = 2.6%/318 yr older than NENA); Western Norway^59^ (mean = 4.9%/560 yr younger than NENA); and Baffin Island/Arctic^60^ (mean = 1.5%/173 yr younger than NENA); see Table S3. We believe that the NENA is the most appropriate for our study site; however, the NENA, Northern Norway and Baffin Bay rates all produce ages within the range of the internal errors. The Western Norway rate is based on data from sites at ~60°N, significantly to the south of Sermilik Fjord. All regional production rates produce ages spanning the YD, meaning our interpretation of the results is not affected by the choice. Density was assumed to be 2.62 g cm^−3^, and we made no correction for inheritance, vegetation or seasonal snow cover, all of which are assumed to be zero or minimal owing to our sampling methods and geomorphic assessment. We assume an erosion rate of zero; using an erosion rate of 2 × 10^−4^cm yr^−1^ increases ages by ~2%^61^.

### Data analysis

Samples were taken in two vertical transects along the fjord (Island transect, samples SF0915-30, and East Wall transect, samples SF0936-55) as well as two additional discrete sampling sites from lower elevations (West Wall site, samples SF0962-64, and Mouth site, samples SF0903/04); comparing the box and whisker plots of the dates from both transects (Fig. S2) shows they have a similar spread of ages, suggesting that the timing and rate of thinning were indistinguishable along the fjord. As such, we are able to combine the samples to form one thinning profile of the ice stream. To test for internal consistency of ages and account for anomalous exposure ages (owing to inheritance, shielding etc.) we first analysed the dataset using the *C_Combine* function in the chronological software OxCal 4.1 (ref.^62^); this performs a chi-square test on bedrock-erratic pairs/triplets, highlighting and discarding the outlier in those pairs/triplets that failed. The function then combines the remaining individual samples within each pair/triplet into one event using a cumulative probability density function. This process led to the exclusion of five samples (Table S2): four bedrock, of which two were anomalously old (SF0924/SF0941) and two anomalously young (SF0916/SF0947); and one erratic, which was anomalously old (SF0903). The 17 samples remaining were re-analysed, together with two radiocarbon dates from basal organic lake sediments on the east wall of Sermilik fjord (Fig. 1 (ref.^26^)) using the Bayesian modelling of the *T_Scaled* analysis function within OxCal 4.1 (ref.^27^). Using Bayes theorem with a Poisson process deposition model, the algorithms employed sample possible solutions with a probability that is the product of the prior and posterior likelihood probabilities. Taking into account the deposition model (i.e. those samples at higher altitudes must be the oldest and vice versa) and the actual ^10^Be measurements, the posterior probability densities quantify the most likely age distributions; the outlier option was used to detect ages that fall outside the calibration model for each group and, where necessary, down-weight their contribution to the final age estimates. In this analysis, none of the remaining samples were considered outliers. This analysis generates the thinning profile of the ice stream shown in Fig. 2A. The Bayesian approach, together with the inclusion of the two radiocarbon dates from basal organic lake sediments, allows the errors on the age envelope to be wrapped down from the typically high errors of the ^10^Be exposure ages, particularly at the lower end of the profile. Of the seventeen ^10^Be exposure ages included in the model, only five were from unpaired events; of these five, only two were bedrock, one of which (SF0925) was younger than its discarded erratic pair, and therefore not experiencing inheritance. Although the remaining unpaired bedrock sample (SF0915, whose erratic pair was anomalously young when compared to other samples of similar heights, and therefore has the potential of inherited ^10^Be) is used to help constrain the upper end of the profile, numerous other samples at this elevation and below (e.g. SF0936/37 (bedrock-erratic), SF0943 (erratic), SF0920/21 (bedrock-erratic)) have ^10^Be exposure age envelopes well within or prior to the onset of the YD. As such, we can be confident that our thinning trajectory is not significantly skewed towards older ages by any potential ^10^Be inheritance within bedrock samples. The ages resulting from the Bayesian modelling in OxCal are shown in Table S4. Our analyses show that deglaciation of the highest sample (423 m a.s.l.) commenced at 13.0 ± 0.4 ka (1.s.d.) (13.05 ± 0.75 ka to 2 s.d.) and that the ice stream thinned to an elevation of 47 m a.s.l. by 10.3 ± 0.2 ka to 1.s.d. (10.95 ± 0.65 ka to 2.s.d.). This is equivalent to an average modelled thinning rate of ~0.14 m yr^−1^, or ~0.23 m yr^−1^ during the Younger Dryas alone (i.e. that due only to non-atmospheric drivers). This is on a comparable order of magnitude to the thinning seen in the fastest-flowing outlet glaciers of Greenland today (~0.84 m yr^−1^ in glaciers flowing faster than 100 m yr^−1^ (ref.^63^)); it is however important to note the difference in observational timescales when making these comparisons.

### Climate modelling

A transient experiment of the last deglaciation was performed with the Earth System model LOVECLIM (ref.^43^). LOVECLIM comprises an ocean general circulation model and a thermodynamic-dynamic sea ice model, with an horizontal resolution of 3° × 3° and 20 vertical levels, coupled to a spectral T21quasi-geostrophic atmospheric model^64^. Starting from the background conditions of the Last Glacial Maximum, the model is forced with the time-varying evolution of solar insolation^31^, ice-sheet topography^65^, high latitude albedo and atmospheric CO_2_ (ref.^66^) for the period 21 ka to 10 ka B.P. The millennial-scale variability of the last deglaciation is simulated by the addition of meltwater in the North Atlantic, Arctic and/or the Southern Ocean. A weakening of the AMOC during the YD is simulated by a 0.25 Sv freshwater flux applied in the Arctic Ocean (175°W-95°W, 67°N-83°N) between 13 and 12.2 ka B.P.

### Data availability statement

The data supporting this manuscript is available in the Supplementary information.

## Electronic supplementary material

Supplementary Information
