# Supplementary material for: Greenland ice mass loss during the Younger Dryas driven by Atlantic Meridional Overturning Circulation feedbacks
Source: Sci Rep. 2018 Aug 9;8:11307. doi: 10.1038/s41598-018-29226-8 (PMC6085367; doi:10.1038/s41598-018-29226-8)
Supplement: Supplementary file 1 — Supplementary Information [file 41598_2018_29226_MOESM1_ESM.docx]

Title: Greenland ice mass loss during the Younger Dryas driven by Atlantic Meridional Overturning Circulation feedbacks

**Authors**

Eleanor Rainsley*^1^, Laurie Menviel^2^, Christopher J. Fogwill^2,3^, Chris S.M. Turney^2^, Anna L. C. Hughes^4^, Dylan H. Rood^5,6^

**Affiliations**

1. School of Geography, Geology and the Environment, University of Keele, Staffordshire, UK
2. Climate Change Research Centre and PANGEA Research Centre, School of Biological, Earth and Environmental Sciences, University of New South Wales, Sydney, NSW 2052, Australia

School of Geography, Geology and Environmental Science, Keele University, Staffordshire, United Kingdom

Department of Earth Science, University of Bergen and Bjerknes Centre for Climate Research, Bergen 5007, Norway

Department of Earth Science and Engineering, Imperial College London, South Kensington Campus, London SW7 2AZ, UK

Scottish Universities Environmental Research Centre, East Kilbride G75 0QF, UK

*****Corresponding author: Eleanor Rainsley, e.b.fogwill@keele.ac.uk School of Geography, Geology and the Environment, University of Keele, Staffordshire, UK

**Supplementary Information**

Table S1. Sample details.

Table S2. Age calculation.

Table S3. Production rate comparison.

Table S4. OxCal chronology

Fig. S1. Raw ^10^Be exposure ages plotted against elevation

Fig. S2. Box and whisker plots of sample age populations for the Island and East wall transects

Fig. S3. Modelled annual mean sea surface temperature

Fig. S4. Modelled precipitation over SE Greenland

Fig. S5. Modelled ocean temperature anomalies

**Supplementary Information**

| **Sample name** | **Latitude (DD)** | **Longitude (DD)** | **Elevation (m a.s.l.)** | **Thickness (cm)** | **Density**  **(g cm^-3^)** | **Shielding^1^** | **Quartz mass (g)** | **^9^Be carrier (mg)** | **^10^Be/^9^Be ratio (10^-13^)** | **Uncertainty (10^-15^)** | **^10^Be**  **(10^4^ at. g^-1^)** | **^10^Be error (10^3^ a.t g^-1^)** | **Blank ratio (10^-15^)** | **Blank error (10^-16^)** |
| --- | --- | --- | --- | --- | --- | --- | --- | --- | --- | --- | --- | --- | --- | --- |
| SF0903* | 66.27978 | -37.72613 | 40 | 6.5 | 2.62 | 0.995 | 32.3399 | 0.2621 | 1.06 | 4.80 | 5.27 | 2.53 | 5.16 | 6.31 |
| SF0904* | 66.27979 | -37.72605 | 47 | 8 | 2.62 | 0.995 | 32.4311 | 0.2629 | 0.87 | 2.71 | 4.30 | 1.46 | 5.16 | 6.31 |
| SF0915* | 66.23833 | -37.58562 | 423 | 12.5 | 2.62 | 0.998 | 19.5668 | 0.2678 | 0.86 | 3.40 | 7.44 | 3.04 | 1.83 | 2.14 |
| SF0916* | 66.238316 | -37.58555 | 427 | 7 | 2.62 | 0.998 | 19.6845 | 0.2702 | 0.77 | 2.69 | 6.64 | 2.43 | 1.83 | 2.14 |
| SF0920 | 66.23358 | -37.58694 | 299 | 3 | 2.62 | 0.991 | 33.7009 | 0.2665 | 1.40 | 4.69 | 7.05 | 2.41 | 1.83 | 2.14 |
| SF0921 | 66.23361 | -37.58686 | 294 | 5 | 2.62 | 0.991 | 72.2166 | 0.2717 | 2.85 | 6.47 | 6.89 | 1.58 | 1.83 | 2.14 |
| SF0924 | 66.2289 | -37.59194 | 178 | 7 | 2.62 | 0.991 | 43.2214 | 0.2685 | 1.72 | 4.88 | 6.81 | 1.97 | 1.83 | 2.14 |
| SF0925 | 66.2289 | -37.59194 | 178 | 5 | 2.62 | 0.991 | 97.6275 | 0.2712 | 3.46 | 7.81 | 6.17 | 1.40 | 1.83 | 2.14 |
| SF0929 | 66.22622 | -37.59279 | 76 | 8.5 | 2.62 | 0.987 | 24.9034 | 0.1862 | 0.93 | 2.32 | 4.82 | 1.32 | 3.65 | 7.41 |
| SF0930 | 66.22625 | -37.59284 | 77 | 5 | 2.62 | 0.987 | 35.1795 | 0.2218 | 1.12 | 3.25 | 4.96 | 1.52 | 3.65 | 7.41 |
| SF0936 | 66.04441 | -37.69803 | 419 | 6 | 2.62 | 0.995 | 20.3527 | 0.27 | 0.85 | 3.65 | 7.14 | 3.16 | 1.83 | 2.14 |
| SF0937 | 66.04447 | -37.69796 | 419 | 11 | 2.62 | 0.995 | 37.6962 | 0.269 | 1.59 | 5.69 | 7.23 | 2.63 | 1.83 | 2.14 |
| SF0941 | 66.04841 | -37.69908 | 306 | 9 | 2.62 | 0.980 | 73.8742 | 0.2723 | 3.33 | 8.04 | 7.89 | 1.92 | 1.83 | 2.14 |
| SF0943 | 66.04859 | -37.69907 | 304 | 6 | 2.62 | 0.993 | 14.3932 | 0.2709 | 0.61 | 2.72 | 7.24 | 3.37 | 1.83 | 2.14 |
| SF0947 | 66.05265 | -37.7088 | 195 | 8.5 | 2.62 | 0.994 | 22.6859 | 0.2701 | 0.69 | 2.64 | 5.16 | 2.07 | 1.83 | 2.14 |
| SF0948 | 66.0563 | -37.70879 | 189 | 6 | 2.62 | 0.994 | 59.3411 | 0.2692 | 2.00 | 4.88 | 5.81 | 1.44 | 1.83 | 2.14 |
| SF0953* | 66.05986 | -37.70488 | 110 | 8.5 | 2.62 | 0.994 | 32.2591 | 0.2145 | 1.12 | 2.93 | 5.21 | 1.45 | 3.65 | 7.41 |
| SF0954* | 66.05988 | -37.70493 | 111 | 10 | 2.62 | 0.994 | 22.2082 | 0.2266 | 0.71 | 2.15 | 4.82 | 1.70 | 5.48 | 8.09 |
| SF0955* | 66.05992 | -37.70486 | 110 | 10 | 2.62 | 0.994 | 26.8037 | 0.2266 | 0.88 | 2.90 | 5.02 | 1.84 | 5.48 | 8.09 |
| SF0962* | 65.85698 | -38.00609 | 112 | 7 | 2.62 | 0.990 | 30.155 | 0.205 | 1.03 | 3.33 | 4.86 | 1.68 | 3.65 | 7.41 |
| SF0963* | 65.85691 | -38.00598 | 110 | 7 | 2.62 | 0.990 | 18.3509 | 0.181 | 0.73 | 3.51 | 4.98 | 2.56 | 3.65 | 7.41 |
| SF0964* | 65.85703 | -38.00618 | 116 | 7.5 | 2.62 | 0.990 | 13.3978 | 0.2266 | 0.51 | 1.98 | 5.60 | 2.61 | 5.48 | 8.09 |

Table S1. Sample details. ^10^Be/^9^Be ratios are uncorrected for background ratios. ^10^Be concentrations are corrected for background ratios and calculated using NIST SRM4325 ^10^Be measurement standard and calibration (*53*) with a reported ^10^Be/^9^Be ratio 2.79x10^-11^ and ^10^Be half-life 1.387 Ma (*52,53*). Zero erosion assumed and standard pressure used. ^1^Ratio of the production rate at the shielded site to that for a 2π surface at the same location calculated using the CRONUS-Earth geometric shielding calculator version 1.1. *Samples previously published in (*21*).

| **Sample name** | **Location** | **Bedrock/erratic** | **Elevation (m a.s.l.)** | **Exposure age (ya)** | **External uncertainty (yrs)** | **Internal uncertainty (yrs)** |
| --- | --- | --- | --- | --- | --- | --- |
| SF0903 | Mouth | Bedrock | 40 | 12700 | 837 | 558 |
| **SF0904** | **Mouth** | **Erratic** | **47** | **10405** | **579** | **280** |
| **SF0915** | **Island** | **Bedrock** | **423** | **12661** | **753** | **429** |
| SF0916 | Island | Bedrock | 427 | 10791 | 610 | 308 |
| **SF0920** | **Island** | **Bedrock** | **299** | **12659** | **724** | **378** |
| **SF0921** | **Island** | **Erratic** | **294** | **12639** | **669** | **263** |
| SF0924 | Island | Erratic | 178 | 14271 | 784 | 363 |
| **SF0925** | **Island** | **Bedrock** | **178** | **12722** | **674** | **267** |
| **SF0929** | **Island** | **Erratic** | **76** | **11415** | **636** | **309** |
| **SF0930** | **Island** | **Bedrock** | **77** | **11419** | **656** | **346** |
| **SF0936** | **East wall** | **Bedrock** | **419** | **11593** | **713** | **432** |
| **SF0937** | **East wall** | **Erratic** | **419** | **12220** | **717** | **398** |
| SF0941 | East wall | Bedrock | 306 | 14869 | 797 | 334 |
| **SF0943** | **East wall** | **Erratic** | **304** | **13185** | **806** | **483** |
| SF0947 | East wall | Bedrock | 195 | 10672 | 618 | 333 |
| **SF0948** | **East wall** | **Erratic** | **189** | **11861** | **632** | **258** |
| **SF0953** | **East wall** | **Bedrock** | **110** | **11786** | **660** | **324** |
| **SF0954** | **East wall** | **Erratic** | **111** | **11015** | **661** | **384** |
| **SF0955** | **East wall** | **Erratic** | **110** | **11475** | **698** | **416** |
| **SF0962** | **West wall** | **Erratic** | **112** | **10848** | **647** | **371** |
| **SF0963** | **West wall** | **Erratic** | **110** | **11144** | **789** | **566** |
| **SF0964** | **West wall** | **Bedrock** | **116** | **12491** | **842** | **576** |

**Table S2. Age calculation**. Exposure age calculated using NENA production rate, assuming no inheritance, zero erosion, density 2.62g cm-3 and standard atmosphere, calculated with the CRONUS-Earth^10^Be-^26^Al exposure age calculator version 2.2 using the time-dependant Lal/Stone scaling scheme. Sample locations as shown on Figure 1. Samples in bold are those used by Oxcal 4.1 to calculate thinning trajectory.

**Table S3. Production rate comparison.** ^10^Be ages calculated using production rates from five different calibration data sets. Mean differences from those produced using the Northeast American (NENA) rate were, respectively: Global: -11.4%; Northern Norway: +2.6%; Western Norway: -4.9%; Baffin Island/Arctic: -1.5%. Model exposure age assuming no inheritance, zero erosion, density 2.62g cm^-3^ and standard atmosphere, calculated using the CRONUS-Earth^10^Be-^26^Al exposure age calculator version 2.2 using the time-dependant Lal/Stone scaling scheme. In this study, the ages calculated with NENA were used, highlighted in grey.

| **Sample name** | **Modelled age (ya)** | **Uncertainty (yrs)** |
| --- | --- | --- |
| SF0904 | 10286 | 192 |
| *K-6038* | *10346* | *185* |
| SF0929/30 | 11127 | 202 |
| *AAR-1542* | *11268* | *149* |
| SF0953/54/55* | 11380 | 163 |
| SF0962/63/64* | 11396 | 170 |
| SF0925 | 11934 | 227 |
| SF0948 | 11999 | 212 |
| SF0920/21* | 12568 | 202 |
| SF0943 | 12611 | 216 |
| SF0936/37* | 13088 | 385 |
| SF0915 | 13122 | 393 |

**Table S4. OxCal chronology.** Chronology developed from OxCal v.4.1 using the Poisson process deposition model^23^. Modelled ages in years ago (ya), uncertainties to 1 standard deviation. Italics denote basal organic lake sediment radiocarbon dates from^57^. *Bedrock-erratic pair/triplet exposure ages combined to a single event by the C-Combine function in Oxcal v4.1^(ref^ *^62^*^)^.

**Fig. S1. Raw ^10^Be exposure ages plotted against elevation.** Island and East wall transects in blue and red respectively, discrete sample sites in green (West wall; samples SF0962-64) and orange (Mouth; samples SF0903/04). Calculated using NENA production rate, Lal/Stone time dependent scaling scheme. Error bars are external uncertainties for each sample (Table S2). Small symbol size denotes outliers. Grey box denotes Younger Dryas.

**Fig. S2. Box and whisker plots of sample age populations for the Island and East wall transects.** Island (blue), East wall (red). The box encloses the area between the first and third quartiles, the horizontal line represents the median, and whiskers show one standard deviation. Samples that lie outside one standard deviation from the mean are shown with a circle. Younger Dryas denoted by grey box.

**Fig. S3. Modelled annual mean sea surface temperature.** Annual mean sea surface temperature (shaded, °C), 0.1 m sea-ice contour (black) and mixed layer depth contours (red, >200 m) as simulated in the transient deglacial experiment*^43^*. (constructed using ferret http://ferret.pmel.noaa.gov/Ferret/).

**Fig. S4. Modelled precipitation over SE Greenland.** Precipitation over SE Greenland (50-W, 61-72°N), simulated in the transient deglacial experiment^43^. (constructed using

ferret http://ferret.pmel.noaa.gov/Ferret/).

**Fig. S5. Modelled ocean temperature anomalies.** Ocean temperature anomalies (shaded) at 484-693m for 12.5-14ka as simulated in the transient deglacial experiment^43^. (constructed using

ferret http://ferret.pmel.noaa.gov/Ferret/).
